# Transcriptomic and Metabolomics Profiling of Phage–Host Interactions between Phage PaP1 and *Pseudomonas aeruginosa*

**DOI:** 10.3389/fmicb.2017.00548

**Published:** 2017-03-30

**Authors:** Xia Zhao, Mengyu Shen, Xingyu Jiang, Wei Shen, Qiu Zhong, Yuhui Yang, Yinling Tan, Melissa Agnello, Xuesong He, Fuquan Hu, Shuai Le

**Affiliations:** ^1^Department of Microbiology, Third Military Medical University, Chongqing, China; ^2^Department of Bioinformatics, Third Military Medical UniversityChongqing, China; ^3^Department of Clinical Laboratory, Xinqiao Hospital, Third Military Medical UniversityChongqing, China; ^4^Department of Clinical Laboratory, Daping Hospital, Third Military Medical UniversityChongqing, China; ^5^School of Dentistry, University of California, Los Angeles, Los AngelesCA, USA

**Keywords:** *Pseudomonas aeruginosa*, bacteriophage, phage–host interaction, transcriptomics, NMR-based metabolomics

## Abstract

The basic biology of bacteriophage–host interactions has attracted increasing attention due to a renewed interest in the therapeutic potential of bacteriophages. In addition, knowledge of the host pathways inhibited by phage may provide clues to novel drug targets. However, the effect of phage on bacterial gene expression and metabolism is still poorly understood. In this study, we tracked phage–host interactions by combining transcriptomic and metabolomic analyses in *Pseudomonas aeruginosa* infected with a lytic bacteriophage, PaP1. Compared with the uninfected host, 7.1% (399/5655) of the genes of the phage-infected host were differentially expressed genes (DEGs); of those, 354 DEGs were downregulated at the late infection phase. Many of the downregulated DEGs were found in amino acid and energy metabolism pathways. Using metabolomics approach, we then analyzed the changes in metabolite levels in the PaP1-infected host compared to un-infected controls. Thymidine was significantly increased in the host after PaP1 infection, results that were further supported by increased expression of a PaP1-encoded thymidylate synthase gene. Furthermore, the intracellular betaine concentration was drastically reduced, whereas choline increased, presumably due to downregulation of the choline–glycine betaine pathway. Interestingly, the choline–glycine betaine pathway is a potential antimicrobial target; previous studies have shown that *betB* inhibition results in the depletion of betaine and the accumulation of betaine aldehyde, the combination of which is toxic to *P. aeruginosa*. These results present a detailed description of an example of phage-directed metabolism in *P. aeruginosa*. Both phage-encoded auxiliary metabolic genes and phage-directed host gene expression may contribute to the metabolic changes observed in the host.

## Introduction

Bacteriophages (phages) are parasites that rely heavily on bacterial host metabolism for replication. To initiate infection, phages bind to receptors on the host cell surface and inject their genetic material into the cell. This allows the phage to redirect the host metabolism for its own replication through host translation of phage-encoded proteins (Labrie et al., 2010). *Pseudomonas aeruginosa* phages are being increasingly studied due to the potential for phage therapy (Gorski et al., 2016). *P. aeruginosa* is an opportunistic pathogen that infects burn wound patients and those with cystic fibrosis (Chatterjee et al., 2016), and is a public health concern due to high rates of multi-drug resistance. Phage therapy is a promising alternative to treating *P. aeruginosa* infections (Citorik et al., 2014; Barbu et al., 2016; Gorski et al., 2016); therefore, a solid understanding of the molecular phage–host interactions will be essential for the regulation and legislation of phage therapy in the near future (Ceyssens and Lavigne, 2010). In addition, cellular processes targeted by phage, such as essential replication or transcription functions, may point to potential antimicrobial drug targets (Liu et al., 2004).

However, understanding of the molecular mechanisms of phage–host interactions mainly comes from studies of model bacteria, such as *Escherichia coli* (Baxter et al., 2006; Belley et al., 2006; Roucourt and Lavigne, 2009), and there is still only limited understanding in other species. A few studies have recently reported findings of phage–host interactions using transcriptomic and metabolomic approaches in *P. aeruginosa*, *Yersinia enterocolitica, Bacillus subtilis*, and the marine microbe *Sulfitobacter* sp. 2047 (Lavigne et al., 2013; Ankrah et al., 2014; Chevallereau et al., 2016; De Smet et al., 2016; Leskinen et al., 2016; Mojardin and Salas, 2016). Chevallereau et al. (2016) used next-generation “omics” approaches to investigate the interactions between *P. aeruginosa* and bacteriophage PAK_P3 and found that RNA processing was hijacked by phage infection and that bacterial transcripts were significantly depleted. De Smet et al. (2016) used high-coverage metabolomics tools to study the metabolic changes of *P. aeruginosa* induced by five different phages. They found that metabolic impacts are highly phage specific; phage-encoded auxiliary metabolic genes (AMGs) reprogram the host metabolism in phage-specific ways (De Smet et al., 2016). By studying the interactions between *P. aeruginosa* and temperate phage PaP3, our group found that the early expressed genes of PaP3 have a strong regulatory effect on host gene expression, particularly genes involved in amino acid metabolism (Zhao et al., 2016b). Furthermore, we found that the phage protein Gp70.1 alters the expression of 178 genes in *P. aeruginosa* and can directly bind to RpoS, a global regulator of broad functions, including biofilm formation and stress response (Zhao et al., 2016a).

Previously, we isolated a *P. aeruginosa* phage from hospital sewage, named PaP1, that belongs to *Myoviridae* (Lu et al., 2013). The goal of the current study was to investigate the interactions between lytic *P. aeruginosa* phage PaP1 (Le et al., 2013) and its host PA1 (Le et al., 2014; Lu et al., 2015) by combining transcriptomic and metabolomic approaches. We found a significant transcriptional change in 6.2% (354/5655) of the host protein-coding genes that were downregulated by PaP1 infection. The significant changes in metabolite concentrations in the PaP1-infected host can be explained by phage-encoded AMGs and phage-directed gene expression.

## Materials and Methods

### Bacterial Strains and Growth Conditions

*Pseudomonas aeruginosa* PA1 (Lu et al., 2015) and lytic phage PaP1 (Lu et al., 2013) were stored in our laboratory at -80°C in glycerol. *P. aeruginosa* was cultured aerobically in Luria-Bertani (LB) medium (5 g yeast extract, 10 g tryptone, 10 g NaCl per liter) at 37°C for all experiments. PaP1 particles were collected and purified using CsCl gradient ultracentrifugation (Lu et al., 2013).

### One-Step Growth Curve

For the one-step growth curve of PaP1, we used a modification of the methods of Lu et al. (2013). The early-logarithmic phase cultures of *P. aeruginosa* (OD600 = 0.2) were infected with phage (multiplicity of infection = 10). After incubation at 37°C for 5 min to allow adsorption, the mixture was centrifuged for 30 s at 13,000 × g. Un-adsorbed phage was removed from the supernatants by washing twice with LB medium. Pellets were resuspended in 5 ml LB, and the cultures were grown at 37°C with shaking at 160 rpm. The total time of adsorption and washing was about 10 min. A total of 50 μL of sample was taken every 10 min, and the number of PaP1 particles was determined using the double-layer agar plaque method. Three biological repeats were performed. The burst time and size were calculated based on the one-step growth curve.

### Microarray Analysis and RT-qPCR

For both microarray analysis and RT-qPCR, total RNA was isolated from four groups of bacterial cultures (0, 5, 15, and 45 min after phage infection) by SV Total RNA Isolation System (Promega, USA, Z3100). The uninfected samples (0 min) served as a control. Microarray hybridization was performed using 5665 custom-designed probes for the *P. aeruginosa* genome and 342 probes for phage PaP1 (Agilent 8 × 15 K array). The intergenic regions were not covered. Raw data from the microarray experiments was deposited in the GEO database with accession number GSE80117. In the RT-qPCR analysis, cDNA synthesis was prepared with PrimeScript RT reagent kit (TaKaRa Bio; Dalian, China) according to the manufacturer’s recommendations. Quantitative real-time PCR was performed using SYBR Premix Ex Taq II (TaKaRa Bio; Dalian, China). Primers used in this study are listed in Supplementary Table S1. The 16S rRNA gene was selected as the reference gene for normalization.

### Extraction of Intracellular Metabolites

Phage-infected bacteria or phage-free bacteria were cultured for 40 min (Supplementary Figure S1). Briefly, 1 L of culture from each group was used for each NMR sample, and five replicate samples were prepared per group. The cultures were placed on ice for 20 min, centrifuged at 15, 000 × *g* for 10 min at 4°C, and the supernatant was removed. The cell pellets were subsequently washed thrice with pre-chilled PBS, followed by centrifugation at 15, 000 × *g* for 5 min at 4°C. All samples were snap frozen in liquid nitrogen and stored at -80°C until NMR analysis. Briefly, 50 mg of freeze-dried cells per sample was weighed and suspended in 1 mL ultrapure water. Cell suspensions were further sonicated on ice for eight cycles of a 4 s on/3 s off cycling program (Sonics VX-130, USA) and then centrifuged for 15 min at 13,000 rpm and 4°C. The cell lysate was filtered through a 3 KDa ultrafiltration membrane (Mimacon, USA) at 13,000 rpm for 45 min at 4°C. Briefly, 50 μl DSS standard solution (Anachro, Canada) was added to 450 μl filtrate per sample, and centrifuged at 13, 000 × *g* at 4°C for 2 min before being transferred to the NMR tubes (Norwell, USA).

### NMR Spectroscopic and Data Analysis

Metabolic profiling and peak identification were performed by Anachro Technologies Inc. (Wuhan, China) using described methods (Weljie et al., 2006; Ouattara et al., 2012). All of the NMR experiments were performed on an Agilent DD2 600 MHz spectrometer equipped with a triple-resonance cryogenic probe at 298.15 K. Metabolites were identified and quantified using Chenomx NMR Suit (version 8.1, Chenomx, Edmonton, AB, Canada). A line broadening of 0.5 Hz was applied to the free induction decay prior to the Fourier transformation, phasing, and baseline correction. Data were then carefully phased and baseline corrected in Chenomx Processor. All spectra were referenced to the internal standard (DSS) and analyzed by experienced analysts against the Chenomx Compound Library. From the 10 spectra, 48 metabolites were identified and quantified. All information on metabolite concentration were exported to excel and normalized by weight across all parallel samples before use in the multivariable analysis. Principal component analysis (PCA) and partial least squares discriminant analysis (PLS-DA) were performed by pcaMethod Bioconductor and pls package in R. ggplot2 in R was used for data visualization. For quantitative metabolomics, raw data files were deposited to the EMBL-EBI MetaboLights database with identifier MTBLS431. The complete dataset can be accessed at the following url: http://www.ebi.ac.uk/metabolights/MTBLS431.

### Data Analysis and Visualization

Microarray data were normalized and analyzed by GeneSpring version GX10.0 (Agilent Technologies). Differentially expressed genes (DEGs; exhibiting at least twofold change in expression) were screened via one-way ANOVA (*p* < 0.05) with Benjamini–Hochberg correction for multiple comparisons (Hochberg and Benjamini, 1990). Gene co-expression analysis was performed with linear regression, which quantified the co-expression of interaction partners based on gene expression similarity of the microarray data sets. Linear correlation (Pearson’s correlation, *cor*) was first computed as described previously (Horvath and Dong, 2008). To make a visual representation, the significant interactions (*P* < 0.001) between phage and host genes were selected to construct the network by Cytoscape (Cline et al., 2007).

In NMR metabolomics, the software MestReNova 9.0 (MestreLab, Santiago de Compostela, Spain) was used to obtain peak areas from the raw spectrum. All information on metabolite concentration was exported to excel and normalized by weight across all parallel samples before use in the multivariable analysis. Principal Components Analysis (PCA) and Partial Least Squares Discriminant Analysis (PLS-DA) were performed by PCA Methods Bioconductor and pls package in R. For each metabolite, the Variable Importance in the Projection (VIP) score was calculated to determine the significantly changed metabolites (VIP score > 1) in phage-infected cells (Chong and Jun, 2005). ggplot2 in R was used for data visualization.

## Results and Discussion

### Influence of Phage Infection on Host Gene Expression

**FIGURE 1:**
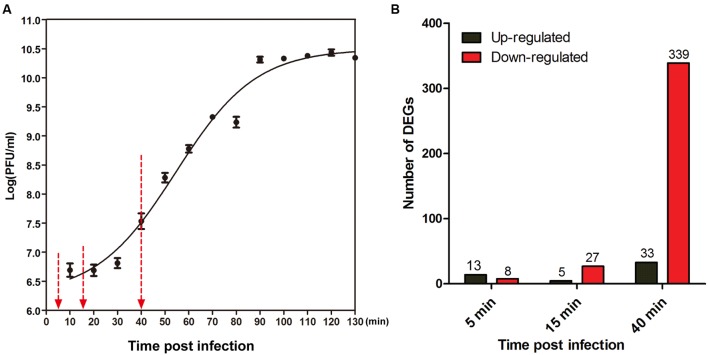
**Global changes induced by PaP1 on host gene expression.** **(A)** One-step growth curve of phage PaP1 in host *P. aeruginosa* PA1. Three replicates were performed. Red arrows indicate the three sampling time points for microarray analysis. **(B)** Number of differentially expressed genes (DEGs) in PaP1-infected PA1 at each time point compared to un-infected PA1.

According to the one-step growth curve of PaP1, the latent time is approximately 20 min, and the burst time is approximately 40 min post-infection (**Figure 1A**). To track the dynamic interaction between phage and host throughout the infection cycle, we examined the transcriptional changes at three time points after phage infection (5, 15, and 40 min) using microarray analysis, with the phage-uninfected host cells (0 min) as the control. These time points correspond to early, middle, and late infection phases, respectively. Supplementary Figure S1 is a simplified scheme of the experimental design.

In three independent experiments, total cellular RNA was extracted from *P. aeruginosa* PA1 cultures uninfected (0 min) or infected with PaP1 for 5, 15, or 40 min and then used for microarray analysis. A total of 5,655 specific probes were designed for the analysis of PA1 gene expression. Compared with the uninfected host, 7.1% (398/5655) of genes of phage-infected host were identified as DEGs (fold change ≥ 2, *p* < 0.05), including 349 downregulated and 50 upregulated genes (**Figure 1B** and Supplementary Table S2). Approximately 85% (339/398) of DEGs were downregulated at the late infection phase, whereas fewer genes were differentially expressed at 5 min and 15 min. This suggests that gene expression of host *P. aeruginosa* was massively suppressed at 40 min after phage infection.

### Specific KEGG Pathways Changed by Phage Infection

**FIGURE 2:**
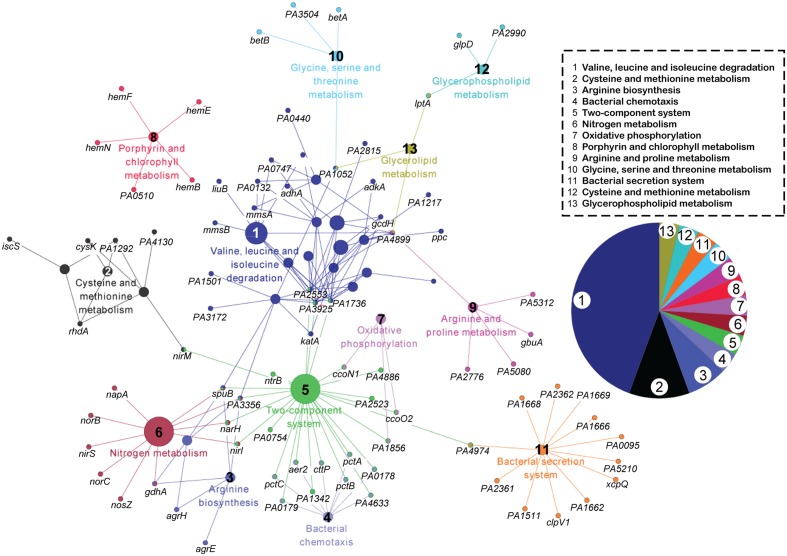
**KEGG pathway network analysis of downregulated genes (*p* < 0.05) in phage-infected PA1.** The functionally grouped network was visualized using Cytoscape based on the degree of connectivity (node size) between pathways and genes (nodes). The 349 downregulated genes at the three time points were involved in 13 pathways, which are distinguished by different colors and numbered 1 to 13. The pie chart displays the corresponding pathways.

First, we specifically focused on the pathways inhibited by phage infection. Kyoto Encyclopedia of Genes and Genomes (KEGG) pathway analysis was performed on the 349 downregulated genes at the three time points (Kanehisa and Goto, 2000). This pathway enrichment analysis based on kappa statistics and visualization was conducted through ClueGO/CluePedia plugin of the Cytoscape software with default parameters (kappa score threshold = 0.4, over view term = Smallest P-Value, sharing group percentage = 50.0) (**Figure 2**) (Bindea et al., 2009). The downregulated genes were concentrated in 13 pathways. The three most observed pathways (valine, leucine, and isoleucine degradation; cysteine and methionine metabolism; and arginine biosynthesis) are related to cellular amino acid metabolism. In addition, bacterial chemotaxis, two-component system, nitrogen metabolism, and oxidative phosphorylation were slightly repressed by phage infection. Thus, the inhibition of amino acid metabolism in the host may be important for phage PaP1 replication.

This pattern of bacterial response is similar to other phage–host studies (Lavigne et al., 2013; Leskinen et al., 2016; Zhao et al., 2016b) in which many host genes are repressed. Only a few DEGs (51, Supplementary Table S2) were upregulated, including membrane proteins involved in energy and small-molecule transport, such as *narK1*, *narH*, *narJ*, and *fptA*.

### Gene Co-expression Analysis of PaP1 and Host

We further explored phage–host interactions on a genome-wide level using gene co-expression analysis of PaP1 and its host. According to the calculation of correlation (*cor*), 169 host genes had significant interactions (*p* < 0.001) with 39 PaP1 genes during PaP1 infection. Remarkably, all 215 gene pairs had *cor* values < 0, indicating a negative interaction. As shown in the gene interaction network (Supplementary Figure S2), the PaP1 genes occupied almost all centers of subnetworks. These results revealed that phage genes play a central and inhibitory role on host gene expression.

**FIGURE 3:**
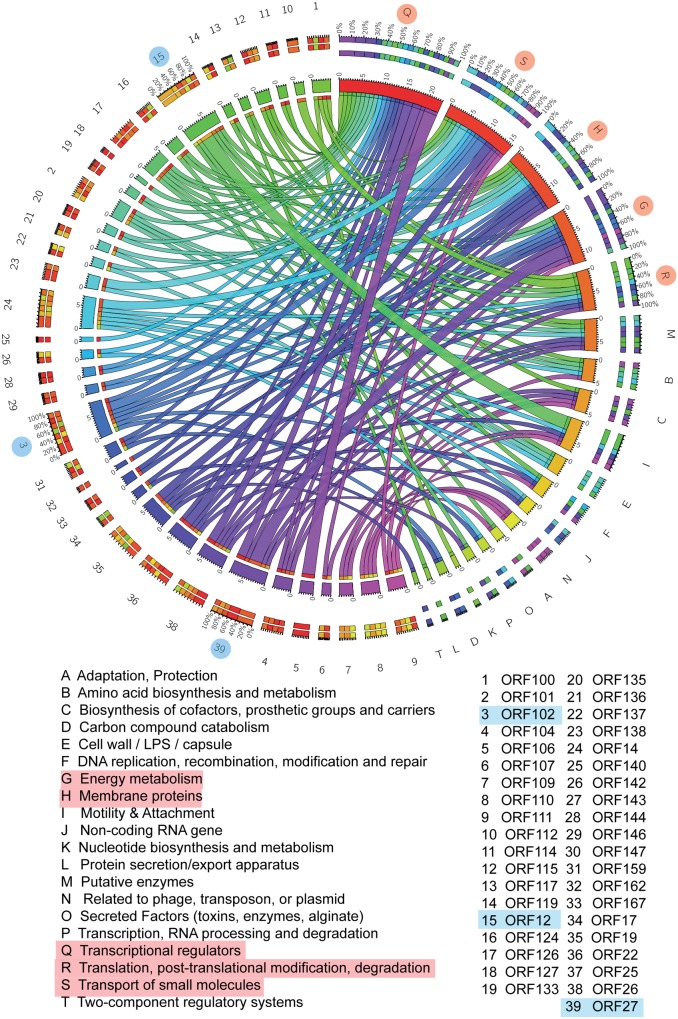
**Gene interaction network between PaP1 and host PA1.** PseudoCAP function analysis of PA1 genes in the gene–gene network. The circos plot, which consists of 20 PseudoCAP functions of the host and 39 phage genes, was constructed based on the gene co-expression network. The PseudoCAP functions are shown with different colors, and the relative size of the links indicates the number of host genes in each functional term; wider links indicate more host genes in that term. The color box for phage genes (the outermost ring) indicates the functional composition of their host gene partners.

To further investigate the biological functions of the host that are modulated by phage infection, *P. aeruginosa* Genome Database (PseudoCAP) function analysis was performed on the 169 host genes involved in the network (Winsor et al., 2005). As shown in the circos plot (**Figure 3**), the top five most enriched PseudoCAP functions included transcriptional regulators; transport of small molecules; membrane proteins; energy metabolism; and translation, posttranslational modification, and degradation.

For the phage genes, both the network map and circos plot revealed that ORF12 had the most host gene partners, followed by ORF102 and ORF27 (Supplementary Figure S2). Gene co-expression network analysis is a powerful approach to detect functional relevance between genes. Genes with a common mediator tend to have a similar expression pattern (Horvath and Dong, 2008). Thus, we inferred that ORF12, ORF102, and ORF27 might be transcriptional regulators during phage infection. To further investigate the impact of these genes on host physiology, studies that include cross-expression of phage genes in the host should be conducted (Zhao et al., 2016a).

The roles of phage proteins on bacterial physiology has been extensively studied (Danovaro et al., 2008; Hurwitz et al., 2013; Ceyssens et al., 2014; Wagemans et al., 2014; Hood and Berger, 2016) and reviewed (Roucourt and Lavigne, 2009) because it is not only an interesting biological phenomenon, but also a rational strategy for identifying potential antimicrobial drug targets (Braff et al., 2016). Liu et al. (2004) found that *Staphylococcus aureus* DnaI is the target of phage protein 77ORF104. By screening small-molecule compounds that inhibit the binding of 77ORF104 to DnaI from commercially available libraries, Liu et al. (2004) identified two compounds that are active against DnaI and have a very low minimum inhibitory concentration against *S. aureus in vitro*. Our group also identified GP70.1 from transcriptomic analysis of phage–*P. aeruginosa* interactions and found that GP70.1 directly binds to the key regulator RpoS to redirect host metabolism (Zhao et al., 2016a). Therefore, further investigation of the interactions of PaP1-encoded proteins ORF102, ORF102, and ORF27 with host targets is necessary.

### Intracellular Metabolite Changes at the Late Infection Phase

**FIGURE 4:**
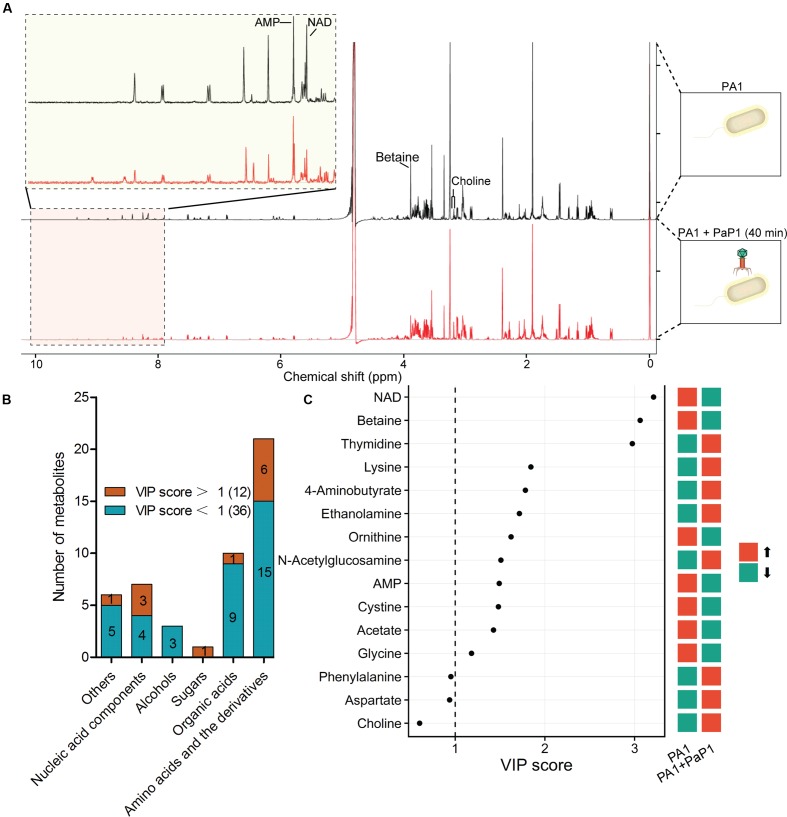
**Metabolomic analysis of phage-infected *P. aeruginosa*.** **(A)** Superimposed 600 MHz ^1^H-NMR spectra of *P. aeruginosa* with or without phage infection. The chemical shift in NMR spectra ranged from 0 to 10 ppm. Significantly changed peaks are marked in the profiles. **(B)** Metabolites identified through NMR spectra. A total of 48 metabolites were detected based on Chenomx database, of which 12 were significantly changed by phage infection (VIP score > 1). **(C)** Variable importance in projection (VIP) analysis is used to show the top 15 changed metabolites with chemical shift. A higher VIP score indicates a more significant difference. Red indicates the metabolite was increased after phage infection, green indicates it was decreased.

Metabolites are downstream products of transcriptional and translational processes and thus provide abundant information about the catalytic and regulatory properties of gene products. A ^1^H-NMR-based metabolomics approach was applied to complement the microarray data and to investigate phage-induced changes of intracellular metabolites at the late infection phase (40 min) compared with the uninfected host (0 min) (**Figure 4A**).

Principal component analysis of the normalized NMR data from 10 samples (five replicate samples) shows a separation between control and phage-infected *P. aeruginosa*, indicating that there were significant changes in metabolites induced by phage infection. Overall, 48 metabolites were identified from the NMR spectra, including 21 amino acids and derivatives, 10 organic acids, 7 nucleic acid components, 3 alcohols, 1 sugar, and 6 other metabolites (**Figure 4B**). Comparison of the metabolite levels in phage-infected bacteria with uninfected samples based on variable importance in projection (VIP) analysis revealed 12 metabolites with significantly altered levels (VIP score > 1) (**Figure 4C**).

The majority of the altered metabolites were amino acids or derivatives, which is consistent with the microarray data. Five out of the 12 significantly altered metabolites, including thymidine, lysine, 4-aminobutyrate, ethanolamine, and *N*-acetylglucose, were increased in the phage-infected samples. The cellular concentrations of NAD^+^ and betaine were the most significantly decreased, with VIP scores >3.

Pathway enrichment analysis was applied in order to investigate the altered metabolic pathways. The results revealed that 48 metabolites detected in the NMR spectra were enriched into 43 KEGG pathways. As shown in Supplementary Figure S3, the significantly changed pathways had a lower *p*-value and higher pathway impact value. The most enriched pathways were involved in the metabolism and biosynthesis of several amino acids, namely, cysteine, methionine, glycine, serine, threonine, phenylalanine, tyrosine, and tryptophan. This finding is consistent with the microarray dataset and indicates that the cellular amino acid metabolism was shifted after phage replication.

### Correlation between Phage-Encoded AMGs and Levels of Host Metabolites

**FIGURE 5:**
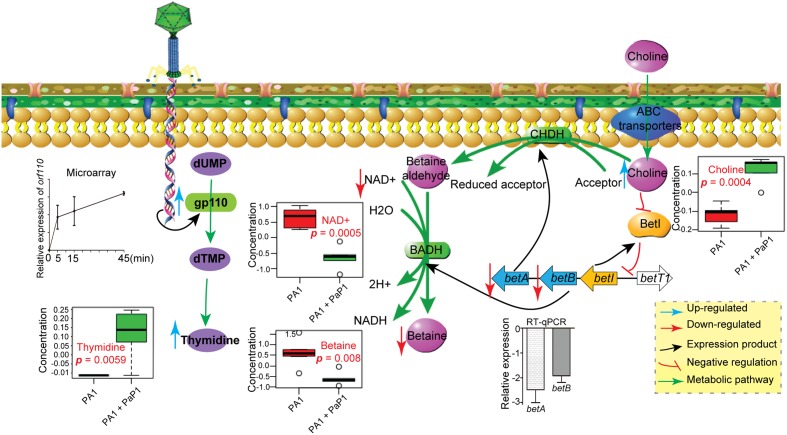
**Model of observed cellular changes in PaP1-infected *P. aeruginosa*.** Choline increased, whereas NAD^+^ and betaine decreased, which is presumably the result of the downregulation of the *betA* and *betB* genes. Thymidine increased, which is most likely linked to the expression of phage *gp110*-encoded thymidylate synthase.

To investigate the cause of metabolic changes in the host, potential AMGs were predicted from the PaP1 genome (accession number HQ832595) (**Table 1**) using BLASTn (Altschul et al., 1990). Interestingly, the PaP1 gene ORF 110 encodes a thymidylate synthase, which is highly expressed during the infection cycle as detected by microarray data and RT-qPCR (**Figure 5**). Thymidine (*p* = 0.0059) was present in significantly higher levels after phage infection (**Figure 5**), which indicates that it may be an essential substrate for *de novo* PaP1 genome synthesis.

**Table 1:** Predicted AMGs in phage PaP1 and phiKZ.

Phage	ORF	Predicted function	E-value	Pathway	Reference
PaP1	32	Deoxycytidylate deaminase	4.00E-33	Nucleotide	Lu et al., 2013
	97	Exodeoxyribonuclease	3.00E-62	Nucleotide	
	110	Thymidylate synthase	4.00E-79	Nucleotide	
phiKZ	4	Dihydrofolate reductase	5.00E-22	Nucleotide	Ankrah et al., 2014
	188	Thymidylate kinase	6.00E-12	Nucleotide	
	214	Deoxycytidine triphosphate deaminase	4.00E-51	Nucleotide	
	235	Thymidylate synthase	4.00E-26	Nucleotide	
	260	dCMP deaminase	3.00E-14	Nucleotide	
	305	Ribonucleotide reductase beta chain	2.00E-16	Nucleotide/glutathione	
	306	Ribonucleotide reductase alpha chain	2.00E-99	Nucleotide/glutathione	


The effect of phage-encoded AMGs on host metabolism was suggested by two other metabolomics studies. Phage phiKZ encodes seven AMGs (**Table 1**), which are involved in the nucleotide synthesis, including a thymidylate synthase (De Smet et al., 2016) Consequently, a significant increase of the molecules of pyrimidine synthesis was detected, such as *N*-carbamoyl-L-aspartate and thymidine. Ankrah et al. (2014) identified viral AMGs from the Global Ocean Survey, which may partially explain the changed metabolites during phage infection, such as amino sugar and nucleotide sugar metabolism.

### Phage-Inhibited Gene Expression on the Changes of Host Metabolites

Although the expression of AMGs may explain some of the changes in host metabolite levels, many others could not be explained, such as the increase in the level of choline and the depletion of NAD^+^ and betaine. We further examined if the observed changes in metabolic pathways were a consequence of differential expression of host genes induced by phage infection. In *P. aeruginosa*, choline is oxidized to betaine through a two-step process catalyzed by choline dehydrogenase (CHDH) and NAD^+^-dependent betaine aldehyde dehydrogenase (BADH), which are encoded by *betA* and *betB*, respectively (Landfald and Strom, 1986; Velasco-Garcia et al., 2006a; Fitzsimmons et al., 2012) (**Figure 5**). As observed from the microarray and RT-qPCR, both *betA* and *betB* were downregulated at 40 min after phage infection, which may inhibit choline catabolism and lead to a decrease in betaine, potentially explaining the metabolic observations in which there was a significant increase in choline (*p* = 0.0004) and decrease in betaine (*p* = 0.008) in the phage-infected *P. aerugino*sa. NAD^+^ acts as a substrate in the reaction catalyzed by BADH, which constitutes the second step of the choline catabolic pathway.

Cellular NAD^+^ was significantly decreased after phage infection (**Figure 4C**), which further inhibits betaine synthesis. The decreased NAD^+^ might reflect downregulated energy and amino acid metabolism in the host; NAD^+^ is an important product of energy metabolic processes. According to the microarray results, a high percentage of downregulated host DEGs are related to energy metabolism. Therefore, phage-induced changes of host metabolic pathways are also mediated through the differential expression of host genes.

Interestingly, inhibition of the choline–glycine betaine pathway is highly toxic to *P. aeruginosa*. Choline is transported into cells through transporters BetT-I and BetT-III and oxidized by CHDH to betaine aldehyde, which is further oxidized to glycine betaine by BADH (Belda et al., 2016). Glycine betaine is an important osmoprotectant that helps *P. aeruginosa* survive during periods of osmotic stress, such as in infected human tissues. Furthermore, betaine aldehyde is highly toxic (Boch et al., 1996; Sage et al., 1997; Velasco-Garcia et al., 2003). Thus, Velasco-Garcia et al. (2003, 2006b) found that disulfiram, a drug used in aversion treatment of alcoholism, can inhibit BADH and could be a potential antimicrobial agent for *P. aeruginosa* infection. Therefore, the mechanism by which phage PaP1 inhibits the choline–glycine betaine pathway should be further investigated, and may provide a novel strategy for antimicrobial drug design (Liu et al., 2004).

## Conclusion

We present an extensive description of phage–host transcriptomic and metabolomic interactions between *P. aerugino*sa and lytic phage PaP1. The metabolic changes observed in the host may result from both phage-encoded AMGs and phage-directed host gene expression. However, the impact of phage-encoded AMGs on host physiology and phage replication needs more extensive experimental testing. Further investigation into the mechanism through which phage can suppress host gene expression may prove crucial for developing novel antibacterial strategies.

## Author Contributions

SL and FH conceived the study. XZ, MS, XJ, and QZ performed the experiments. WS, YY, YT, and SL analyzed the sequence data. SL, XZ, XH, and MA wrote the paper.

## Conflict of Interest Statement

The authors declare that the research was conducted in the absence of any commercial or financial relationships that could be construed as a potential conflict of interest.
