# Supplementary material for: Transcriptomic and Metabolomics Profiling of Phage–Host Interactions between Phage PaP1 and Pseudomonas aeruginosa
Source: Front Microbiol. 2017 Mar 30;8:548. doi: 10.3389/fmicb.2017.00548 (PMC5377924; doi:10.3389/fmicb.2017.00548)
Supplement: Supplementary file 1 [file Data_Sheet_1.DOCX]

**Table S1. Primers for Real-Time qPCR**

| Primer | Primer sequence |
| --- | --- |
| betA-F | ATGCTGAGACCGCTTTCCAC |
| betA-R | ATCCACCACTCGCAGTCCTT |
| betB-F | CGGCATCTGCTGGATCAATA |
| betB-R | AGCTCCACTTGCACGGACTT |
| gp110-F | CCGAGTCACATAGGGAACAT |
| gp110-R | TTACCGCTACGGAACTGGAT |
| 16S rRNA-F | CAAAACTACTGAGCTAGAGTACG |
| 16S rRNA-R | TAAGATCTCAAGGATCCCAACGGCT |

**Table S2. Differentially expressed genes during phage PaP1 infection.**

**Differentially expressed genes at 5 min**

| Locus.Tag | pvalues | foldchange | Gene.Name | PseudoCAP.Function.Class |
| --- | --- | --- | --- | --- |
| PA3874 | 0.026286 | 5.284208233 | narH | Energy metabolism |
| PA3873 | 0.029411 | 4.62237518 | narJ | Energy metabolism |
| ORF42 | 0.020147 | 2.865909721 | NA | NA |
| PA1402 | 0.047829 | 2.551458698 | NA | "Hypothetical, unclassified, unknown" |
| PA1602 | 0.033177 | 2.468594519 | NA | Carbon compound catabolism |
| PA2571 | 0.006711 | 2.297298879 | NA | Two-component regulatory systems |
| PA1888 | 0.04861 | 2.266920698 | NA | "Hypothetical, unclassified, unknown" |
| PA5319 | 0.023808 | 2.174901475 | radC | "DNA replication, recombination, modification and repair" |
| PA5127 | 0.030827 | 2.150090502 | NA | Putative enzymes |
| PA3416 | 0.032579 | 2.135433776 | NA | Energy metabolism |
| PA0788 | 0.040605 | 2.103531937 | NA | "Hypothetical, unclassified, unknown" |
| PA5353 | 0.041284 | 2.068646669 | glcF | Central intermediary metabolism ; Carbon compound catabolism |
| PA2012 | 0.033354 | 2.062407248 | liuD | Carbon compound catabolism |
| PA1163 | 0.029298 | 2.055289379 | NA | Putative enzymes ; Antibiotic resistance and susceptibility |
| PA2581 | 0.033343 | 0.499818255 | NA | "Hypothetical, unclassified, unknown" |
| PA4371 | 0.035967 | 0.470398208 | NA | "Hypothetical, unclassified, unknown" |
| PA0659 | 0.045977 | 0.437390033 | NA | Membrane proteins |
| PA5023 | 0.049091 | 0.419049868 | NA | "Hypothetical, unclassified, unknown" |
| PA0089 | 0.043389 | 0.38766556 | NA | "Hypothetical, unclassified, unknown" |
| PA1844 | 0.003838 | 0.314584627 | NA | "Hypothetical, unclassified, unknown" |
| PA0045 | 0.002915 | 0.294779311 | NA | "Hypothetical, unclassified, unknown" |
| PA3814 | 0.041842 | 0.157613048 | iscS | "Amino acid biosynthesis and metabolism ;  Biosynthesis of cofactors, prosthetic groups and carriers" |

**Differentially expressed genes at 15 min**

| Locus.Tag | pvalues | foldchange | Gene.Name | PseudoCAP.Function.Class | |  |  |  |
| --- | --- | --- | --- | --- | --- | --- | --- | --- |
| PA3877 | 0.013565 | 18.23328 | narK1 | Membrane proteins ; Transport of small molecules | | | | |
| PA3870 | 0.047078 | 5.815456 | moaA1 | "Biosynthesis of cofactors, prosthetic groups and carriers" | | | | |
| PA2007 | 0.044853 | 4.286131 | maiA | Carbon compound catabolism | |  |  |  |
| PA2008 | 0.042693 | 3.793031 | fahA | Carbon compound catabolism | |  |  |  |
| PA5160.1 | 0.045166 | 2.591164 | NA | Non-coding RNA gene | |  |  |  |
| PA0126 | 0.023523 | 0.489042 | NA | "Hypothetical, unclassified, unknown" | | | |  |
| PA2194 | 0.009347 | 0.458 | hcnB | Central intermediary metabolism | | |  |  |
| PA3727 | 0.049575 | 0.446247 | NA | "Hypothetical, unclassified, unknown" | | | |  |
| PA0096 | 0.022365 | 0.439492 | NA | "Hypothetical, unclassified, unknown" | | | |  |
| PA2581 | 0.027719 | 0.417566 | NA | "Hypothetical, unclassified, unknown" | | | |  |
| PA1511 | 0.01315 | 0.412182 | NA | "Hypothetical, unclassified, unknown" | | | |  |
| PA1668 | 0.014457 | 0.40162 | NA | "Hypothetical, unclassified, unknown" | | | |  |
| PA1669 | 0.005238 | 0.391111 | NA | "Hypothetical, unclassified, unknown ; Membrane proteins" | | | | |
| PA3021 | 0.011554 | 0.384426 | NA | "Hypothetical, unclassified, unknown" | | | |  |
| PA1657 | 0.023931 | 0.366885 | NA | "Hypothetical, unclassified, unknown" | | | |  |
| PA0095 | 0.046496 | 0.364298 | NA | "Hypothetical, unclassified, unknown" | | | |  |
| PA0659 | 0.000425 | 0.364145 | NA | Membrane proteins |  |  |  |  |
| PA0088 | 0.035271 | 0.362536 | NA | "Hypothetical, unclassified, unknown" | | | |  |
| PA3729 | 0.007922 | 0.360827 | NA | "Hypothetical, unclassified, unknown" | | | |  |
| PA0097 | 0.000763 | 0.357566 | NA | "Hypothetical, unclassified, unknown" | | | |  |
| PA0089 | 0.035573 | 0.328554 | NA | "Hypothetical, unclassified, unknown" | | | |  |
| PA0087 | 0.011839 | 0.32677 | NA | "Hypothetical, unclassified, unknown" | | | |  |
| PA5054 | 0.009376 | 0.322013 | hslU | Chaperones & heat shock proteins | | |  |  |
| PA0086 | 0.017419 | 0.318801 | NA | "Hypothetical, unclassified, unknown" | | | |  |
| PA0090 | 0.042187 | 0.289243 | clpV1 | "Translation, post-translational modification, degradation ;  Chaperones & heat shock proteins ; Protein secretion/export apparatus" | | | | |
| PA2662 | 0.041617 | 0.195031 | NA | Membrane proteins |  |  |  |  |
| PA0522 | 0.029922 | 0.194808 | NA | "Hypothetical, unclassified, unknown" | | | |  |
| PA5023 | 0.006057 | 0.193269 | NA | "Hypothetical, unclassified, unknown" | | | |  |
| PA0084 | 0.012395 | 0.187929 | NA | "Hypothetical, unclassified, unknown" | | | |  |
| PA2663 | 0.009392 | 0.15452 | ppyR | Membrane proteins ; Cell wall / LPS / capsule | | | | |
| PA3814 | 0.020795 | 0.111083 | iscS | "Amino acid biosynthesis and metabolism ; Biosynthesis of cofactors,  prosthetic groups and carriers" | | | | |
| PA2664 | 0.025965 | 0.048717 | fhp | Energy metabolism |  |  |  |  |

**Differentially expressed genes at 40min**

| Locus.Tag | pvalues | foldchange | Gene.Name | PseudoCAP.Function.Class |
| --- | --- | --- | --- | --- |
| PA2912 | 0.009852 | 7.05247605 | NA | Transport of small molecules |
| PA4221 | 0.031504 | 6.532962959 | fptA | Transport of small molecules |
| PA4815 | 0.037205 | 6.367461186 | NA | Membrane proteins |
| PA1924 | 0.041349 | 6.278032274 | NA | "Hypothetical, unclassified, unknown" |
| PA4226 | 0.009406 | 5.911539002 | pchE | "Secreted Factors (toxins, enzymes, alginate) ; Transport of small molecules" |
| PA5181 | 0.00157 | 4.964288615 | NA | Putative enzymes |
| PA4989 | 0.00123 | 4.604244216 | NA | Transcriptional regulators |
| PA2008 | 0.028413 | 4.505383053 | fahA | Carbon compound catabolism |
| PA4821 | 0.039888 | 4.232110553 | NA | Membrane proteins ; Transport of small molecules |
| PA0156 | 0.039789 | 3.895731135 | triA | Membrane proteins ; Antibiotic resistance and susceptibility |
| PA1329 | 0.047923 | 3.250340376 | NA | "Hypothetical, unclassified, unknown" |
| PA4930 | 0.04201 | 3.215592806 | alr | Amino acid biosynthesis and metabolism |
| PA3721 | 0.018273 | 3.156824874 | nalC | Transcriptional regulators ; Antibiotic resistance and susceptibility |
| PA2408 | 0.032091 | 2.934244397 | NA | Transport of small molecules |
| PA2840 | 0.031865 | 2.892346437 | NA | "Transcription, RNA processing and degradation" |
| PA4635 | 0.010566 | 2.542801363 | NA | "Hypothetical, unclassified, unknown" |
| PA5125 | 0.0347 | 2.525174994 | ntrC | Transcriptional regulators ; Two-component regulatory systems |
| PA1884 | 0.024774 | 2.454412937 | NA | Transcriptional regulators |
| PA0405 | 0.048056 | 2.408084735 | NA | "Hypothetical, unclassified, unknown" |
| PA2241 | 0.023866 | 2.397338023 | pslK | Membrane proteins ; Cell wall / LPS / capsule |
| PA4709 | 0.003193 | 2.394790689 | NA | Putative enzymes |
| PA4754 | 0.010189 | 2.361258487 | NA | Membrane proteins |
| PA1300 | 0.025586 | 2.353795097 | NA | Transcriptional regulators |
| PA0930 | 0.048133 | 2.18908539 | NA | Transport of small molecules ; Two-component regulatory systems |
| PA1137 | 0.005662 | 2.188316425 | NA | Putative enzymes |
| PA0223 | 0.043278 | 2.172801539 | NA | Cell wall / LPS / capsule ; Amino acid biosynthesis and metabolism |
| PA3576 | 0.037256 | 2.158008261 | NA | "Hypothetical, unclassified, unknown" |
| PA0017 | 0.012636 | 2.101211265 | NA | "Hypothetical, unclassified, unknown" |
| PA0802 | 0.00584 | 2.092115105 | NA | Membrane proteins |
| PA5415 | 0.008084 | 2.085303838 | glyA1 | Amino acid biosynthesis and metabolism |
| PA0501 | 0.02414 | 2.0676872 | bioF | "Biosynthesis of cofactors, prosthetic groups and carriers" |
| PA3561 | 0.024121 | 2.049343962 | fruK | Central intermediary metabolism ; Transport of small molecules |
| PA3936 | 0.037053 | 2.033610375 | NA | Membrane proteins ; Transport of small molecules |
| PA0120 | 0.028227 | 0.499938373 | NA | Transcriptional regulators |
| PA0126 | 0.026 | 0.49968555 | NA | "Hypothetical, unclassified, unknown" |
| PA3687 | 0.013865 | 0.499658481 | ppc | Energy metabolism ; Central intermediary metabolism |
| PA1139 | 0.017624 | 0.496458567 | NA | "Hypothetical, unclassified, unknown" |
| PA1835 | 0.022887 | 0.496411501 | NA | "Hypothetical, unclassified, unknown" |
| PA2484 | 0.01371 | 0.496387671 | NA | "Hypothetical, unclassified, unknown" |
| PA4899 | 0.035611 | 0.496358161 | NA | Putative enzymes |
| PA3172 | 0.022482 | 0.495505665 | NA | Putative enzymes |
| PA1052 | 0.046114 | 0.49512233 | NA | "Hypothetical, unclassified, unknown" |
| PA5352 | 0.02726 | 0.490393634 | NA | "Hypothetical, unclassified, unknown" |
| PA3233 | 0.021971 | 0.487570288 | NA | "Hypothetical, unclassified, unknown" |
| PA4186 | 0.047278 | 0.487229077 | NA | "Hypothetical, unclassified, unknown" |
| PA3504 | 0.011641 | 0.487133799 | NA | Putative enzymes |
| PA1501 | 0.02212 | 0.486818444 | NA | "Hypothetical, unclassified, unknown" |
| PA0338 | 0.047736 | 0.486713235 | NA | "Hypothetical, unclassified, unknown ; Motility & Attachment" |
| PA3617 | 0.043503 | 0.482131169 | recA | "DNA replication, recombination, modification and repair" |
| PA5188 | 0.015079 | 0.480793919 | NA | Putative enzymes |
| PA4600 | 0.007868 | 0.47663341 | nfxB | Transcriptional regulators |
| PA0440 | 0.042292 | 0.476287874 | NA | Putative enzymes |
| PA1986 | 0.043869 | 0.474634833 | pqqB | "Biosynthesis of cofactors, prosthetic groups and carriers" |
| PA5507 | 0.020363 | 0.474293493 | NA | "Hypothetical, unclassified, unknown" |
| PA4446 | 0.040698 | 0.473989229 | algW | "Translation, post-translational modification, degradation ; Adaptation, Protection ; Secreted Factors (toxins, enzymes, alginate)" |
| PA0269 | 0.004514 | 0.473096604 | NA | "Hypothetical, unclassified, unknown" |
| PA1188 | 0.025932 | 0.468518509 | NA | Putative enzymes |
| PA0132 | 0.000983 | 0.467538105 | NA | Amino acid biosynthesis and metabolism |
| PA0750 | 0.043844 | 0.466152254 | ung | "DNA replication, recombination, modification and repair" |
| PA4886 | 0.025437 | 0.46436755 | NA | Two-component regulatory systems |
| PA2014 | 0.027752 | 0.463707339 | liuB | Carbon compound catabolism |
| PA5105 | 0.021304 | 0.460578731 | hutC | Transcriptional regulators |
| PA5330 | 0.038877 | 0.459560166 | NA | "Hypothetical, unclassified, unknown" |
| PA5122 | 0.042482 | 0.459088066 | NA | "Hypothetical, unclassified, unknown" |
| PA2680 | 0.042905 | 0.457828754 | NA | Energy metabolism |
| PA1859 | 0.014729 | 0.45727687 | NA | Transcriptional regulators |
| PA4633 | 0.006628 | 0.456775834 | NA | "Adaptation, Protection ; Chemotaxis" |
| PA5197 | 0.002944 | 0.454783556 | rimK | "Transcription, RNA processing and degradation ; Translation, post-translational modification, degradation" |
| PA2889 | 0.021567 | 0.453686794 | atuD | Putative enzymes |
| PA4588 | 0.024366 | 0.451721267 | gdhA | Amino acid biosynthesis and metabolism |
| PA4956 | 0.02676 | 0.45052875 | rhdA | Central intermediary metabolism |
| PA5104 | 0.032342 | 0.449674708 | NA | "Hypothetical, unclassified, unknown" |
| PA3678 | 0.02834 | 0.44917769 | NA | Transcriptional regulators |
| PA1378 | 0.021501 | 0.449043753 | NA | "Hypothetical, unclassified, unknown" |
| PA1217 | 0.038029 | 0.448333305 | NA | Amino acid biosynthesis and metabolism |
| PA2159 | 0.019471 | 0.445813887 | NA | "Hypothetical, unclassified, unknown" |
| PA5161 | 0.024926 | 0.441719149 | rmlB | Carbon compound catabolism ; Cell wall / LPS / capsule |
| PA1727 | 0.046441 | 0.440677242 | mucR | Motility & Attachment ; Cell wall / LPS / capsule |
| PA2826 | 0.021741 | 0.440330206 | NA | "Adaptation, Protection" |
| PA3475 | 0.043743 | 0.43896313 | pheC | "Adaptation, Protection ; Amino acid biosynthesis and metabolism" |
| PA1252 | 0.008255 | 0.438026797 | NA | Putative enzymes |
| PA4657 | 0.013897 | 0.43360093 | NA | "Hypothetical, unclassified, unknown" |
| PA5186 | 0.047838 | 0.431741484 | NA | Putative enzymes |
| PA4027 | 0.021343 | 0.430868498 | NA | "Hypothetical, unclassified, unknown" |
| PA5275 | 0.027931 | 0.421251499 | NA | "Hypothetical, unclassified, unknown" |
| PA5331 | 0.04302 | 0.419979698 | pyrE | Nucleotide biosynthesis and metabolism |
| PA1542 | 0.025637 | 0.418650638 | NA | "Hypothetical, unclassified, unknown" |
| PA1292 | 0.021448 | 0.417554881 | NA | Putative enzymes |
| PA0446 | 0.00892 | 0.41412902 | NA | "Hypothetical, unclassified, unknown" |
| PA3584 | 0.03407 | 0.412767348 | glpD | Central intermediary metabolism ; Energy metabolism |
| PA1344 | 0.017929 | 0.411787906 | NA | Putative enzymes |
| PA5034 | 0.023648 | 0.40963576 | hemE | "Biosynthesis of cofactors, prosthetic groups and carriers" |
| PA1269 | 0.042821 | 0.40700224 | NA | Transcriptional regulators |
| PA0832 | 0.038405 | 0.406805059 | NA | "Hypothetical, unclassified, unknown" |
| PA1733 | 0.015908 | 0.406676405 | NA | "Hypothetical, unclassified, unknown" |
| PA5312 | 0.043335 | 0.403880002 | NA | Putative enzymes |
| PA2897 | 0.023303 | 0.403598725 | NA | Transcriptional regulators |
| PA0337 | 0.030197 | 0.402189656 | ptsP | Transport of small molecules |
| PA0341 | 0.042929 | 0.402042872 | lgt | "Translation, post-translational modification, degradation ; Fatty acid and phospholipid metabolism" |
| PA0298 | 0.027608 | 0.400599113 | spuB | Putative enzymes |
| PA5232 | 0.022622 | 0.399639289 | NA | "Hypothetical, unclassified, unknown" |
| PA1517 | 0.010127 | 0.399624461 | NA | "Hypothetical, unclassified, unknown" |
| PA4831 | 0.027987 | 0.396664347 | NA | Transcriptional regulators |
| PA0418 | 0.038271 | 0.396147935 | NA | "Hypothetical, unclassified, unknown" |
| PA3830 | 0.032736 | 0.396068357 | NA | Transcriptional regulators |
| PA1631 | 0.048871 | 0.395981499 | NA | Putative enzymes |
| PA5263 | 0.048616 | 0.394758031 | argH | Amino acid biosynthesis and metabolism |
| PA3692 | 0.000285 | 0.394329268 | lptF | Membrane proteins ; Transport of small molecules |
| PA0447 | 0.016612 | 0.392101853 | gcdH | Fatty acid and phospholipid metabolism ; Amino acid biosynthesis and metabolism ; Carbon compound catabolism |
| PA4360 | 0.02451 | 0.391633513 | NA | "Hypothetical, unclassified, unknown" |
| PA0607 | 0.040705 | 0.390397905 | rpe | Energy metabolism |
| PA4369 | 0.009003 | 0.390279828 | NA | "Hypothetical, unclassified, unknown" |
| PA2787 | 0.049096 | 0.389584317 | cpg2 | Central intermediary metabolism |
| PA1537 | 0.049419 | 0.389043708 | NA | Putative enzymes |
| PA4434 | 0.032718 | 0.384364927 | NA | Putative enzymes |
| PA1106 | 0.002427 | 0.384132824 | NA | "Hypothetical, unclassified, unknown" |
| PA2849 | 0.013094 | 0.383291983 | NA | Transcriptional regulators |
| PA2802 | 0.048338 | 0.382436487 | NA | Transcriptional regulators |
| PA1539 | 0.02325 | 0.381151038 | NA | "Hypothetical, unclassified, unknown" |
| PA4974 | 0.030499 | 0.379083472 | NA | Protein secretion/export apparatus |
| PA3034 | 0.043532 | 0.377216086 | NA | Transcriptional regulators |
| PA5488 | 0.022347 | 0.375684676 | NA | "Hypothetical, unclassified, unknown" |
| PA3084 | 0.037239 | 0.375098157 | NA | "Hypothetical, unclassified, unknown" |
| PA1172 | 0.046919 | 0.374190422 | napC | Energy metabolism |
| PA2812 | 0.024298 | 0.374185912 | NA | Transport of small molecules |
| PA3570 | 0.005248 | 0.373533368 | mmsA | Amino acid biosynthesis and metabolism ; Carbon compound catabolism |
| PA5372 | 0.021551 | 0.372652475 | betA | "Amino acid biosynthesis and metabolism ; Adaptation, Protection" |
| PA0821 | 0.010991 | 0.372637955 | NA | "Hypothetical, unclassified, unknown" |
| PA1836 | 0.013706 | 0.372462636 | NA | Transcriptional regulators |
| PA4539 | 0.019157 | 0.371575978 | NA | "Hypothetical, unclassified, unknown" |
| PA0289 | 0.046663 | 0.3694323 | gpuR | Transcriptional regulators |
| PA3127 | 0.033091 | 0.369402036 | NA | "Hypothetical, unclassified, unknown" |
| PA4518 | 0.048445 | 0.368742939 | NA | "Hypothetical, unclassified, unknown" |
| PA2830 | 0.029878 | 0.367318879 | htpX | "Adaptation, Protection" |
| PA5155 | 0.030587 | 0.367213697 | NA | Membrane proteins ; Transport of small molecules |
| PA5056 | 0.02663 | 0.364337907 | phaC1 | Central intermediary metabolism |
| PA4330 | 0.031269 | 0.364084346 | NA | Putative enzymes |
| PA0147 | 0.02137 | 0.363957787 | NA | Putative enzymes |
| PA5358 | 0.035591 | 0.363626247 | ubiA | "Energy metabolism ; Biosynthesis of cofactors, prosthetic groups and carriers" |
| PA2703 | 0.014597 | 0.363623764 | NA | "Hypothetical, unclassified, unknown" |
| PA1738 | 0.04307 | 0.361373678 | NA | Transcriptional regulators |
| PA1765 | 0.006306 | 0.360031597 | NA | "Hypothetical, unclassified, unknown" |
| PA1627 | 0.012044 | 0.357996256 | NA | Transcriptional regulators |
| PA3922 | 0.004026 | 0.357976619 | NA | "Hypothetical, unclassified, unknown" |
| PA1308 | 0.024062 | 0.357432969 | NA | Membrane proteins |
| PA2958 | 0.044835 | 0.356046147 | NA | "Hypothetical, unclassified, unknown" |
| PA0005 | 0.043961 | 0.356005035 | lptA | Fatty acid and phospholipid metabolism |
| PA3973 | 0.012159 | 0.353247872 | NA | Transcriptional regulators |
| PA1307 | 0.045468 | 0.349924374 | NA | "Hypothetical, unclassified, unknown" |
| PA3017 | 0.017453 | 0.349614279 | NA | "Hypothetical, unclassified, unknown" |
| PA2248 | 0.036249 | 0.349342154 | bkdA2 | Amino acid biosynthesis and metabolism |
| PA4198 | 0.000358 | 0.349212008 | NA | Putative enzymes |
| PA3091 | 0.041948 | 0.349132241 | NA | "Hypothetical, unclassified, unknown" |
| PA2867 | 0.040888 | 0.348471312 | NA | "Adaptation, Protection ; Chemotaxis" |
| PA4307 | 0.047765 | 0.348118777 | pctC | "Adaptation, Protection ; Chemotaxis" |
| PA5373 | 0.005646 | 0.346303621 | betB | "Amino acid biosynthesis and metabolism ; Adaptation, Protection" |
| PA1621 | 0.036065 | 0.345915422 | NA | Putative enzymes |
| PA0295 | 0.040443 | 0.344553284 | NA | Transport of small molecules |
| PA5206 | 0.032336 | 0.343208228 | argE | Amino acid biosynthesis and metabolism |
| PA5346 | 0.036327 | 0.341971402 | sadB | NA |
| PA5080 | 0.043826 | 0.341730431 | NA | "Translation, post-translational modification, degradation" |
| PA0275 | 0.020469 | 0.341282653 | NA | Transcriptional regulators |
| PA4841 | 0.036651 | 0.340254332 | NA | "Hypothetical, unclassified, unknown" |
| PA3311 | 0.020922 | 0.339562323 | NA | Membrane proteins |
| PA4362 | 0.019201 | 0.338835936 | NA | "Hypothetical, unclassified, unknown" |
| PA3790 | 0.012486 | 0.338582966 | oprC | Transport of small molecules |
| PA3874 | 0.021885 | 0.337757212 | narH | Energy metabolism |
| PA2075 | 0.013161 | 0.336724535 | NA | "Hypothetical, unclassified, unknown" |
| PA2717 | 0.00524 | 0.336593067 | cpo | Central intermediary metabolism |
| PA3587 | 0.024291 | 0.336161942 | metR | Transcriptional regulators |
| PA1314 | 0.037045 | 0.335972819 | NA | "Hypothetical, unclassified, unknown" |
| PA4893 | 0.013829 | 0.333949053 | ureG | "Biosynthesis of cofactors, prosthetic groups and carriers" |
| PA1174 | 0.010505 | 0.332636804 | napA | Energy metabolism |
| PA2362 | 0.008074 | 0.332117351 | NA | "Hypothetical, unclassified, unknown" |
| PA2776 | 0.021905 | 0.330017351 | NA | "Hypothetical, unclassified, unknown" |
| PA5158 | 0.017099 | 0.325627377 | NA | Transport of small molecules |
| PA0180 | 0.00466 | 0.3243647 | NA | "Adaptation, Protection ; Chemotaxis" |
| PA3920 | 0.017093 | 0.32301118 | NA | Membrane proteins ; Transport of small molecules |
| PA3238 | 0.026042 | 0.322703202 | NA | "Hypothetical, unclassified, unknown" |
| PA5433 | 0.026816 | 0.322695835 | NA | "Hypothetical, unclassified, unknown" |
| PA0090 | 0.044259 | 0.319048951 | clpV1 | "Translation, post-translational modification, degradation ; Chaperones & heat shock proteins ; Protein secretion/export apparatus" |
| PA1941 | 0.022123 | 0.316684514 | NA | "Hypothetical, unclassified, unknown" |
| PA2990 | 0.007474 | 0.316402264 | NA | Central intermediary metabolism |
| PA4353 | 0.024857 | 0.315478925 | NA | "Hypothetical, unclassified, unknown" |
| PA0086 | 0.012609 | 0.315068534 | NA | "Hypothetical, unclassified, unknown" |
| PA1421 | 0.022357 | 0.313865046 | gbuA | Amino acid biosynthesis and metabolism |
| PA5027 | 0.003824 | 0.311706386 | NA | "Hypothetical, unclassified, unknown" |
| PA1662 | 0.000322 | 0.309886954 | NA | Putative enzymes |
| PA2195 | 0.007646 | 0.306185913 | hcnC | Central intermediary metabolism |
| PA0836 | 0.03623 | 0.305428455 | ackA | Putative enzymes |
| PA4947 | 0.046047 | 0.305423288 | amiB | Cell wall / LPS / capsule |
| PA0176 | 0.004743 | 0.30376443 | aer2 | "Adaptation, Protection ; Chemotaxis" |
| PA0754 | 0.048496 | 0.302862586 | NA | "Hypothetical, unclassified, unknown" |
| PA4522 | 0.040141 | 0.302690429 | ampD | Cell wall / LPS / capsule |
| PA3056 | 0.036008 | 0.301589778 | NA | "Hypothetical, unclassified, unknown" |
| PA2848 | 0.041069 | 0.29954265 | NA | Transcriptional regulators |
| PA1189 | 0.046079 | 0.298996299 | NA | "Hypothetical, unclassified, unknown" |
| PA2171 | 0.031651 | 0.29749209 | NA | "Hypothetical, unclassified, unknown" |
| PA4101 | 0.014964 | 0.296301448 | bfmR | Transcriptional regulators ; Two-component regulatory systems ; Cell wall / LPS / capsule |
| PA0087 | 0.013951 | 0.295835625 | NA | "Hypothetical, unclassified, unknown" |
| PA1657 | 0.015474 | 0.295047449 | NA | "Hypothetical, unclassified, unknown" |
| PA2633 | 0.03558 | 0.291803409 | NA | "Related to phage, transposon, or plasmid" |
| PA0779 | 0.024348 | 0.288747809 | NA | Putative enzymes |
| PA1484 | 0.041555 | 0.28618356 | NA | Transcriptional regulators |
| PA2471 | 0.020863 | 0.282698176 | NA | "Hypothetical, unclassified, unknown" |
| PA2815 | 0.003607 | 0.279395483 | NA | Putative enzymes |
| PA1849 | 0.002872 | 0.277975874 | NA | "Hypothetical, unclassified, unknown" |
| PA2821 | 0.038923 | 0.277093264 | NA | Central intermediary metabolism |
| PA0517 | 0.010769 | 0.276976916 | nirC | "Biosynthesis of cofactors, prosthetic groups and carriers ; Energy metabolism" |
| PA3872 | 2.73E-05 | 0.276739273 | narI | Energy metabolism |
| PA4961 | 0.030359 | 0.275332623 | NA | Membrane proteins |
| PA2475 | 0.011142 | 0.27509151 | NA | "Adaptation, Protection ; Putative enzymes" |
| PA5124 | 0.047147 | 0.27495175 | ntrB | Two-component regulatory systems |
| PA2194 | 0.020814 | 0.274785955 | hcnB | Central intermediary metabolism |
| PA2523 | 0.044628 | 0.271636989 | NA | Transcriptional regulators ; Two-component regulatory systems |
| PA0744 | 0.029715 | 0.271615891 | NA | Putative enzymes |
| PA1544 | 0.033316 | 0.270855664 | anr | Transcriptional regulators |
| PA2711 | 0.008263 | 0.270229351 | NA | Transport of small molecules |
| PA2120 | 0.035022 | 0.267812648 | NA | "Hypothetical, unclassified, unknown" |
| PA3871 | 0.008552 | 0.266336089 | NA | "Translation, post-translational modification, degradation ; Chaperones & heat shock proteins" |
| PA1067 | 0.023962 | 0.266193399 | NA | Transcriptional regulators |
| PA1064 | 0.026103 | 0.263639704 | NA | "Hypothetical, unclassified, unknown" |
| PA1754 | 0.004677 | 0.263491439 | cysB | Amino acid biosynthesis and metabolism ; Transcriptional regulators |
| PA5378 | 0.023904 | 0.260219961 | NA | "Hypothetical, unclassified, unknown" |
| PA0178 | 0.038316 | 0.259686047 | NA | "Chemotaxis ; Adaptation, Protection ; Two-component regulatory systems" |
| PA3569 | 0.003175 | 0.258372612 | mmsB | Carbon compound catabolism |
| PA2854 | 0.018606 | 0.257640655 | NA | "Hypothetical, unclassified, unknown" |
| PA3021 | 0.005322 | 0.255804272 | NA | "Hypothetical, unclassified, unknown" |
| PA3105 | 0.035197 | 0.254896366 | xcpQ | Protein secretion/export apparatus |
| PA1669 | 0.005334 | 0.254821611 | NA | "Hypothetical, unclassified, unknown ; Membrane proteins" |
| PA1550 | 0.047907 | 0.252601824 | NA | "Hypothetical, unclassified, unknown" |
| PA3250 | 0.013471 | 0.251695769 | NA | "Hypothetical, unclassified, unknown" |
| PA4130 | 0.007873 | 0.251483225 | NA | Central intermediary metabolism |
| PA1826 | 0.022461 | 0.248564829 | NA | Transcriptional regulators |
| PA4542 | 0.042682 | 0.244525434 | clpB | "Translation, post-translational modification, degradation" |
| PA1666 | 0.004631 | 0.243698669 | NA | "Hypothetical, unclassified, unknown" |
| PA1664 | 0.006505 | 0.243391221 | NA | "Hypothetical, unclassified, unknown" |
| PA4079 | 0.04366 | 0.242815734 | NA | Putative enzymes |
| PA0872 | 0.040024 | 0.242616968 | phhA | Amino acid biosynthesis and metabolism |
| PA3356 | 0.044569 | 0.242464748 | NA | "Hypothetical, unclassified, unknown" |
| PA2581 | 0.047245 | 0.241269686 | NA | "Hypothetical, unclassified, unknown" |
| PA2709 | 0.026268 | 0.240582108 | cysK | Amino acid biosynthesis and metabolism |
| PA4842 | 0.004657 | 0.239048604 | NA | "Hypothetical, unclassified, unknown" |
| PA1833 | 0.012026 | 0.237620283 | NA | Putative enzymes |
| PA3925 | 0.048672 | 0.235957879 | NA | Putative enzymes |
| PA3753 | 0.017256 | 0.2346162 | NA | Transport of small molecules |
| PA3723 | 0.005489 | 0.232912535 | NA | Putative enzymes |
| PA5178 | 0.034801 | 0.231782066 | NA | "Hypothetical, unclassified, unknown" |
| PA0743 | 0.008792 | 0.230204823 | NA | Carbon compound catabolism |
| PA5243 | 0.012347 | 0.229815288 | hemB | "Biosynthesis of cofactors, prosthetic groups and carriers" |
| PA2361 | 0.036757 | 0.229625217 | NA | "Hypothetical, unclassified, unknown" |
| PA3615 | 0.041844 | 0.228363695 | NA | "Hypothetical, unclassified, unknown" |
| PA3923 | 0.001613 | 0.227536905 | NA | "Hypothetical, unclassified, unknown" |
| PA2553 | 0.033093 | 0.226327619 | NA | Putative enzymes |
| PA3251 | 0.005998 | 0.224711736 | NA | "Hypothetical, unclassified, unknown" |
| PA0387 | 0.046388 | 0.224592069 | NA | "Hypothetical, unclassified, unknown" |
| PA2771 | 0.038572 | 0.223640134 | NA | "Hypothetical, unclassified, unknown" |
| PA2504 | 0.011506 | 0.223328381 | NA | "Hypothetical, unclassified, unknown" |
| PA5159 | 0.045527 | 0.220081476 | NA | Transport of small molecules ; Antibiotic resistance and susceptibility |
| PA4793 | 0.045728 | 0.219640825 | NA | "Hypothetical, unclassified, unknown" |
| PA0659 | 0.033776 | 0.219314098 | NA | Membrane proteins |
| PA3764 | 0.040012 | 0.218508531 | NA | "Hypothetical, unclassified, unknown" |
| PA3440 | 0.046831 | 0.21808314 | NA | "Hypothetical, unclassified, unknown" |
| PA1668 | 0.041248 | 0.217307416 | NA | "Hypothetical, unclassified, unknown" |
| PA0023 | 0.025701 | 0.212235336 | qor | Energy metabolism |
| PA0406 | 0.04293 | 0.209323528 | NA | "Hypothetical, unclassified, unknown" |
| PA5210 | 0.043735 | 0.208601039 | NA | Protein secretion/export apparatus |
| PA1811 | 0.035153 | 0.20825857 | NA | Transport of small molecules |
| PA0082 | 0.020361 | 0.203664679 | NA | "Hypothetical, unclassified, unknown" |
| PA2557 | 0.04449 | 0.202306578 | NA | Fatty acid and phospholipid metabolism |
| PA4611 | 0.041853 | 0.201722257 | NA | "Hypothetical, unclassified, unknown" |
| PA2073 | 0.024493 | 0.199700382 | NA | Transport of small molecules |
| PA2663 | 0.01002 | 0.199203406 | ppyR | Membrane proteins ; Cell wall / LPS / capsule |
| PA3205 | 0.047288 | 0.198511481 | NA | "Hypothetical, unclassified, unknown" |
| PA0097 | 0.042299 | 0.197928031 | NA | "Hypothetical, unclassified, unknown" |
| PA4384 | 0.035519 | 0.197813837 | NA | "Hypothetical, unclassified, unknown" |
| PA3397 | 0.026153 | 0.197729714 | fpr | "Biosynthesis of cofactors, prosthetic groups and carriers ; Energy metabolism" |
| PA3795 | 0.019561 | 0.194771192 | NA | Putative enzymes |
| PA1976 | 0.0139 | 0.192602644 | ercS' | Two-component regulatory systems |
| PA0732 | 0.040106 | 0.191384045 | NA | "Hypothetical, unclassified, unknown" |
| PA0747 | 0.019546 | 0.19025106 | NA | Putative enzymes |
| PA4761 | 0.008359 | 0.189673513 | dnaK | "DNA replication, recombination, modification and repair ; Adaptation, Protection ; Chaperones & heat shock proteins" |
| PA1003 | 0.045963 | 0.189161775 | mvfR | "Biosynthesis of cofactors, prosthetic groups and carriers ; Transcriptional regulators" |
| PA1342 | 0.049013 | 0.187997541 | NA | Transport of small molecules |
| PA3394 | 0.034511 | 0.187402121 | nosF | Energy metabolism ; Transport of small molecules |
| PA4129 | 0.035459 | 0.18486099 | NA | "Hypothetical, unclassified, unknown" |
| PA3477 | 0.028714 | 0.183896774 | rhlR | "Adaptation, Protection ; Transcriptional regulators" |
| PA4296 | 0.016063 | 0.181170629 | pprB | Transcriptional regulators ; Two-component regulatory systems ; Antibiotic resistance and susceptibility |
| PA0084 | 0.020341 | 0.177157991 | NA | "Hypothetical, unclassified, unknown" |
| PA2718 | 0.023017 | 0.174302548 | NA | Transcriptional regulators |
| PA1736 | 0.041367 | 0.172343023 | NA | Putative enzymes |
| PA3126 | 0.032415 | 0.168427581 | ibpA | Chaperones & heat shock proteins |
| PA0588 | 0.000798 | 0.166656241 | NA | "Hypothetical, unclassified, unknown" |
| PA0307 | 0.008683 | 0.166383601 | NA | "Hypothetical, unclassified, unknown" |
| PA4310 | 0.0462 | 0.163484158 | pctB | "Adaptation, Protection ; Chemotaxis" |
| PA0083 | 0.023332 | 0.160410995 | NA | "Hypothetical, unclassified, unknown" |
| PA4309 | 0.043642 | 0.159128551 | pctA | "Adaptation, Protection ; Chemotaxis" |
| PA4026 | 0.018251 | 0.154575631 | NA | Putative enzymes |
| PA1856 | 0.039718 | 0.152619573 | NA | Putative enzymes |
| PA1658 | 0.032137 | 0.151178184 | NA | "Hypothetical, unclassified, unknown" |
| PA0483 | 0.040508 | 0.149586319 | NA | Putative enzymes |
| PA0092 | 0.040938 | 0.148823107 | NA | "Hypothetical, unclassified, unknown" |
| PA3812 | 0.024765 | 0.148652273 | iscA | "Biosynthesis of cofactors, prosthetic groups and carriers" |
| PA1831 | 0.022523 | 0.146510278 | NA | "Hypothetical, unclassified, unknown" |
| PA2707 | 0.006886 | 0.145251611 | NA | "Hypothetical, unclassified, unknown" |
| PA3041 | 0.007106 | 0.143215362 | NA | Membrane proteins |
| PA3813 | 0.023779 | 0.135486061 | iscU | "Biosynthesis of cofactors, prosthetic groups and carriers" |
| PA5054 | 0.018768 | 0.133702448 | hslU | Chaperones & heat shock proteins |
| PA3911 | 0.003534 | 0.130730206 | NA | "Hypothetical, unclassified, unknown" |
| PA1789 | 0.04075 | 0.12583097 | NA | "Hypothetical, unclassified, unknown" |
| PA1665 | 0.038319 | 0.125014152 | NA | "Hypothetical, unclassified, unknown" |
| PA0388 | 0.016138 | 0.12031443 | NA | "Hypothetical, unclassified, unknown" |
| PA1554 | 0.042298 | 0.120190774 | ccoN1 | Energy metabolism ; Energy metabolism |
| PA1051 | 0.009356 | 0.119094561 | NA | Membrane proteins ; Transport of small molecules |
| PA3912 | 0.038716 | 0.115410477 | NA | "Hypothetical, unclassified, unknown" |
| PA0746 | 0.028538 | 0.114986074 | NA | Putative enzymes |
| PA3465 | 0.033726 | 0.113839084 | NA | Membrane proteins |
| PA1660 | 0.029456 | 0.11327419 | NA | "Hypothetical, unclassified, unknown" |
| PA4387 | 0.048027 | 0.109813111 | NA | Membrane proteins |
| PA3811 | 0.049532 | 0.107278303 | hscB | Chaperones & heat shock proteins |
| PA3815 | 0.037386 | 0.103697036 | NA | "Hypothetical, unclassified, unknown" |
| PA2788 | 0.004506 | 0.102129342 | NA | "Adaptation, Protection ; Chemotaxis" |
| PA0141 | 0.043119 | 0.099812398 | NA | "Hypothetical, unclassified, unknown" |
| PA1556 | 0.04232 | 0.099734212 | ccoO2 | Energy metabolism ; Energy metabolism |
| PA0522 | 0.009185 | 0.098093581 | NA | "Hypothetical, unclassified, unknown" |
| PA0867 | 0.034064 | 0.097306 | mliC | "Hypothetical, unclassified, unknown ; Adaptation, Protection" |
| PA4352 | 0.023924 | 0.096409178 | NA | "Hypothetical, unclassified, unknown" |
| PA3913 | 0.049296 | 0.096368229 | NA | Putative enzymes |
| PA2662 | 0.006115 | 0.096284346 | NA | Membrane proteins |
| PA1596 | 0.011768 | 0.091480752 | htpG | Chaperones & heat shock proteins |
| PA4863 | 0.037657 | 0.090279759 | NA | "Hypothetical, unclassified, unknown" |
| PA5023 | 0.003924 | 0.085935889 | NA | "Hypothetical, unclassified, unknown" |
| PA1546 | 0.031559 | 0.084138937 | hemN | "Biosynthesis of cofactors, prosthetic groups and carriers" |
| PA0515 | 0.013457 | 0.080052307 | NA | "Biosynthesis of cofactors, prosthetic groups and carriers ; Energy metabolism ; Transcriptional regulators" |
| PA0516 | 0.009147 | 0.07959006 | nirF | "Energy metabolism ; Biosynthesis of cofactors, prosthetic groups and carriers" |
| PA1551 | 0.026317 | 0.078958907 | NA | Energy metabolism |
| PA5053 | 0.024446 | 0.072868171 | hslV | Chaperones & heat shock proteins |
| PA4610 | 0.006394 | 0.072849083 | NA | "Hypothetical, unclassified, unknown" |
| PA0512 | 0.028036 | 0.071636952 | NA | "Biosynthesis of cofactors, prosthetic groups and carriers ; Hypothetical, unclassified, unknown ; Energy metabolism" |
| PA0510 | 0.012669 | 0.07158419 | NA | "Biosynthesis of cofactors, prosthetic groups and carriers ; Energy metabolism" |
| PA3393 | 0.007288 | 0.071427089 | nosD | Energy metabolism |
| PA4607 | 0.027591 | 0.071114008 | NA | "Hypothetical, unclassified, unknown" |
| PA5427 | 0.036207 | 0.069821387 | adhA | Energy metabolism ; Carbon compound catabolism |
| PA4389 | 0.039531 | 0.068620996 | NA | Putative enzymes ; Amino acid biosynthesis and metabolism |
| PA0918 | 0.006283 | 0.068202652 | NA | Energy metabolism |
| PA4762 | 0.031194 | 0.066735576 | grpE | "DNA replication, recombination, modification and repair ; Chaperones & heat shock proteins" |
| PA0179 | 0.023125 | 0.06562489 | NA | "Chemotaxis ; Adaptation, Protection ; Two-component regulatory systems" |
| PA4236 | 0.010381 | 0.064031261 | katA | "Adaptation, Protection" |
| PA4463 | 0.044764 | 0.063459572 | NA | "Hypothetical, unclassified, unknown" |
| PA0024 | 0.019843 | 0.055672604 | hemF | "Biosynthesis of cofactors, prosthetic groups and carriers" |
| PA4067 | 0.047411 | 0.054850765 | oprG | Membrane proteins |
| PA0655 | 0.023157 | 0.05194627 | NA | "Hypothetical, unclassified, unknown" |
| PA3814 | 0.009925 | 0.051125452 | iscS | "Amino acid biosynthesis and metabolism ; Biosynthesis of cofactors, prosthetic groups and carriers" |
| PA4578 | 0.032729 | 0.043660861 | NA | "Hypothetical, unclassified, unknown" |
| PA3392 | 0.001808 | 0.042245549 | nosZ | Energy metabolism |
| PA4922 | 0.018107 | 0.039845503 | azu | Energy metabolism |
| PA0511 | 0.002961 | 0.035419189 | nirJ | "Biosynthesis of cofactors, prosthetic groups and carriers ; Energy metabolism" |
| PA1746 | 0.01012 | 0.030846297 | NA | "Hypothetical, unclassified, unknown" |
| PA4587 | 0.009769 | 0.027091404 | ccpR | Energy metabolism |
| PA2381 | 0.030193 | 0.02626195 | NA | "Hypothetical, unclassified, unknown" |
| PA0526 | 0.030172 | 0.023663441 | NA | "Hypothetical, unclassified, unknown" |
| PA1847 | 0.037469 | 0.021562832 | NA | "Hypothetical, unclassified, unknown" |
| PA2664 | 0.004062 | 0.020971554 | fhp | Energy metabolism |
| PA0525 | 0.004093 | 0.01979658 | NA | Energy metabolism |
| PA1123 | 0.012356 | 0.017634528 | NA | "Hypothetical, unclassified, unknown" |
| PA0520 | 0.006341 | 0.015057145 | nirQ | Energy metabolism ; Central intermediary metabolism |
| PA0509 | 0.002559 | 0.012081064 | nirN | "Biosynthesis of cofactors, prosthetic groups and carriers ; Energy metabolism" |
| PA0521 | 0.017361 | 0.010292269 | NA | Energy metabolism |
| PA0519 | 0.005541 | 0.009471121 | nirS | Energy metabolism |
| PA0518 | 0.006175 | 0.008774536 | nirM | "Biosynthesis of cofactors, prosthetic groups and carriers ; Energy metabolism" |
| PA0524 | 0.003148 | 0.003336499 | norB | Energy metabolism |
| PA0523 | 0.006037 | 0.001106883 | norC | Energy metabolism |
