# Supplementary material for: Transcriptomic and Metabolomics Profiling of Phage–Host Interactions between Phage PaP1 and Pseudomonas aeruginosa
Source: Front Microbiol. 2017 Mar 30;8:548. doi: 10.3389/fmicb.2017.00548 (PMC5377924; doi:10.3389/fmicb.2017.00548)

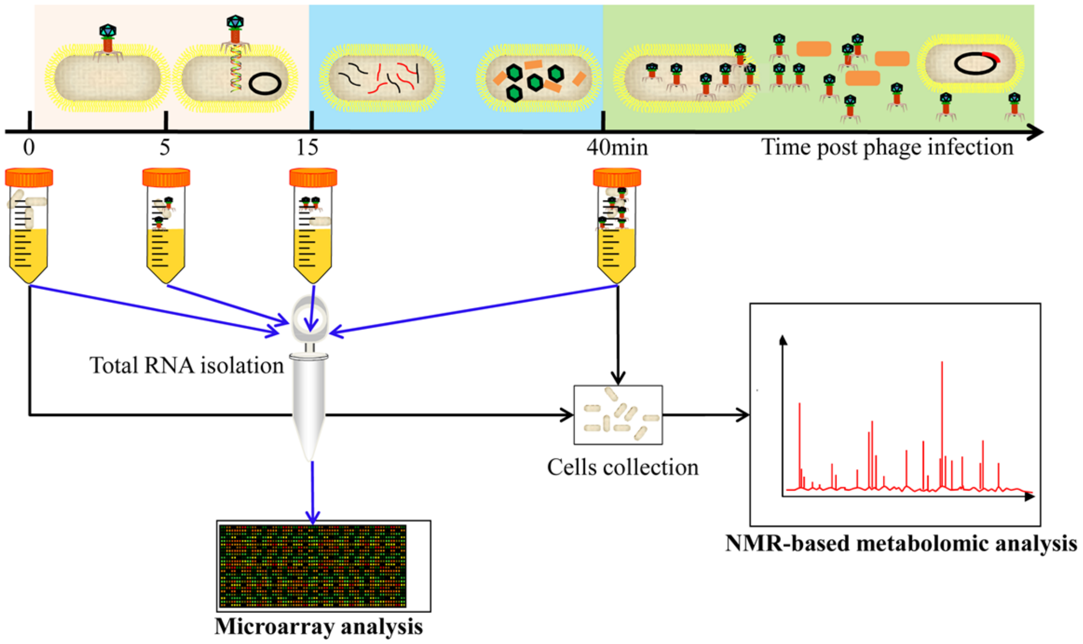

Figure S1. A simplified scheme of the experimental design. Total RNA was isolated from 4 groups (prior to phage infection, and 5, 15, and 45 min after phage infection). NMR samples were collected from two groups (prior to phage infection and 45 min after phage infection).

Figure S2. Gene–gene interaction network of PaP1 and PA1. A node and edge in the network represents the gene and interaction between genes, respectively. The number of the interactions of each gene was weighted by degree from 1 to 12, which are shown with gradient color and size. Genes with higher numbers of interactions are shown in a larger size.

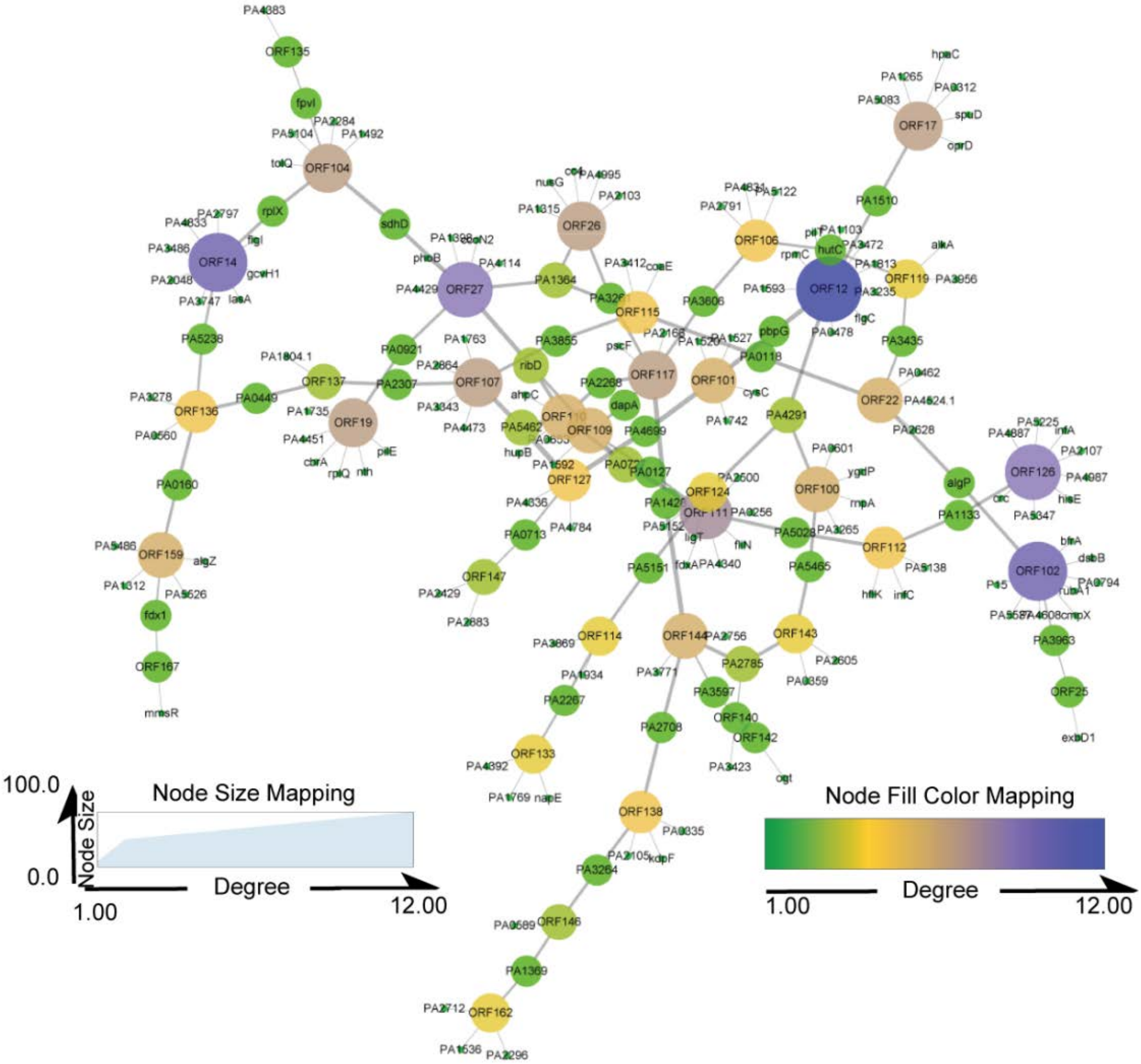

Figure S3. Metabolic pathways enrichment analysis of metabolomics data. The detected 48 metabolites in the NMR analysis were involved with 43 KEGG pathways. Influence of phage infection on the metabolic pathways of the host was weighted by statistical *p*-values and pathway impact values. The more significantly changed pathway had a higher -log (*p*) value (lower *p* value, orange) and pathway impact value (bigger circle). The top 4 most affected pathways by phage infection were marked with detailed names: “Cysteine and methionine metabolism”, “Glycine, serine and threonine metabolism”, “Nicotinate and nicotinamide metabolism” and “Phenylalanine, tyrosine and tryptophan biosynthesis”.

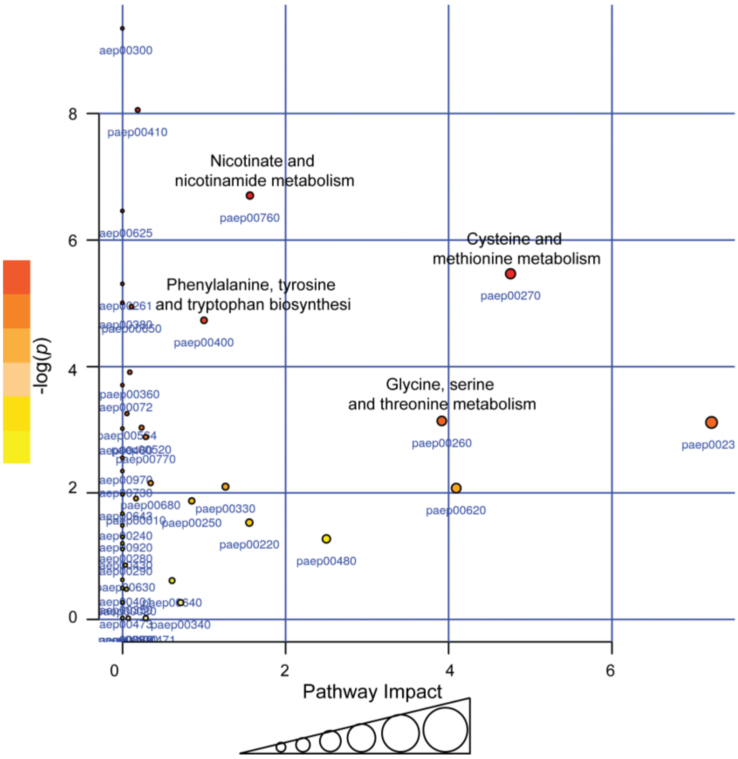

Supplement: Supplementary file 2 [file Image_1.pdf]
